# The Sun, Moon, Wind, and Biological Imperative–Shaping Contrasting Wintertime Migration and Foraging Strategies of Adult Male and Female Northern Fur Seals (*Callorhinus ursinus*)

**DOI:** 10.1371/journal.pone.0093068

**Published:** 2014-04-10

**Authors:** Jeremy T Sterling, Alan M. Springer, Sara J. Iverson, Shawn P. Johnson, Noel A. Pelland, Devin S. Johnson, Mary-Anne Lea, Nicholas A. Bond

**Affiliations:** 1 National Marine Mammal Laboratory, Alaska Fisheries Science Center, National Marine Fisheries Service, NOAA, Seattle, WA, United States of America; 2 Institute of Marine Science, University of Alaska Fairbanks, Fairbanks, AK, United States of America; 3 Department of Biology, Dalhousie University, Halifax, Nova Scotia, Canada; 4 Johnson Veterinary Service, The Marine Mammal Center, Sausalito, CA, United States of America; 5 School of Oceanography, University of Washington, Seattle, WA, United States of America; 6 Institute for Marine and Antarctic Studies, University of Tasmania, Hobart, Australia; 7 University of Washington/JISAO, Seattle, WA, United States of America; Texas A&M University-Corpus Christi, United States of America

## Abstract

Adult male and female northern fur seals (*Callorhinus ursinus*) are sexually segregated in different regions of the North Pacific Ocean and Bering Sea during their winter migration. Explanations for this involve interplay between physiology, predator-prey dynamics, and ecosystem characteristics, however possible mechanisms lack empirical support. To investigate factors influencing the winter ecology of both sexes, we deployed five satellite-linked conductivity, temperature, and depth data loggers on adult males, and six satellite-linked depth data loggers and four satellite transmitters on adult females from St. Paul Island (Bering Sea, Alaska, USA) in October 2009. Males and females migrated to different regions of the North Pacific Ocean: males wintered in the Bering Sea and northern North Pacific Ocean, while females migrated to the Gulf of Alaska and California Current. Horizontal and vertical movement behaviors of both sexes were influenced by wind speed, season, light (sun and moon), and the ecosystem they occupied, although the expression of the behaviors differed between sexes. Male dive depths were aligned with the depth of the mixed layer during daylight periods and we suspect this was the case for females upon their arrival to the California Current. We suggest that females, because of their smaller size and physiological limitations, must avoid severe winters typical of the northern North Pacific Ocean and Bering Sea and migrate long distances to areas of more benign environmental conditions and where prey is shallower and more accessible. In contrast, males can better tolerate often extreme winter ocean conditions and exploit prey at depth because of their greater size and physiological capabilities. We believe these contrasting winter behaviors 1) are a consequence of evolutionary selection for large size in males, important to the acquisition and defense of territories against rivals during the breeding season, and 2) ease environmental/physiological constraints imposed on smaller females.

## Introduction

Body size commonly affects animal behavior and, in many pinniped species, sexual dimorphism results in habitat segregation and reduced intraspecific competition for resources [1]–[4]. Sexual selection or ecological divergence or both may explain the differences in size between males and females [3]. Larger size generally requires greater resources to meet energy requirements, but in pinnipeds it also allows for a greater capacity to dive longer, exploit resources at deeper depths, and endure harsher environmental conditions [5]–[7]. However, the necessity to dive deeper for food depends on the depth of preferred prey, which is affected by prey behaviors and a variety of environmental factors such as bathymetric and oceanographic gradients, high wind speeds due to winter storms, lunar periodicity, the day-night cycle, and seasonality in production [8]–[21].

While size predicts aerobic capacity and dive performance across pinniped species, factors affecting local foraging conditions also can cause inter- and intraspecific differences in diving and movement behavior [7], [22]–[24]. For example, Antarctic and New Zealand fur seals (*Arctocephalus gazella* and *A. forsteri*) and northern and southern elephant seals (*Mirounga angustirostris* and *M. leonina*) show differences between sexes in the location and depth of foraging [1], [2], [22], [25]. Adult males of both species of fur seals are much larger, dive deeper, and forage in different habitats than their female counterparts [1], [2], [26]. However, in both northern and southern elephant seals, body mass predicts mean dive durations, but not mean dive depths. And, contrary to predictions based on the size differential between males and females, northern elephant seal females in some cases display longer mean dive durations than the much larger adult males [23], [24]. These characteristics are explained by their foraging behavior along migration routes–adult male northern elephant seals migrate to and along the continental margins of western North America and undertake benthic dives to similar depths as females foraging farther offshore, which, in contrast, access the deep scattering layer while in pelagic waters [23], [25]. Generally for both fur seals and elephant seals, larger size allows for longer, and in some cases, deeper dives; however, the interplay between predator, prey, and habitat varies and, as noted by Staniland and Robinson [2], the manner in which their behavior is expressed (i.e., dive durations, depths, and foraging routes) is dependent on the local environment and its effect on prey resources.

In polygynous species like elephant seals and fur seals, the large size of males confers advantages in fasting and holding territories to defend females against rivals during the breeding season, and thus strengthens males’ reproductive potential [11]. Adult male northern fur seals (NFS; *Callorhinus ursinus*) may weigh as much as 350 kg, much more than adult females (30–50 kg), and have not been observed migrating into the same regions of the North Pacific Ocean [11], [16], [27]–[32]. Generally, the assumption has been that contrasting physiological and dive capabilities, due to sexual size dimorphism, combined with differences in prey distribution and abundance among ecosystems, lead to the divergent wintering areas. However, there has been limited empirical evidence to support this assumption.

Given that variability in seal movement and diving can be species, size, and habitat specific, acquiring spatial and temporal biological and physical measurements relevant at individual and group scales is crucial to aid our understanding of broader scale observations of intraspecific, interannual, and decadal variability in behavior, and ultimately of population dynamics. In this study, we examined the migratory behavior of adult female and male NFS from St. Paul Island (Bering Sea, Alaska, USA), where overall abundance has declined by 70% in the past few decades for uncertain reasons [33]. Previous studies summarized winter distribution and dive behavior for females [16] and winter distribution only for males [32]. Those studies were not conducted simultaneously, leaving open the possibility of interannual physical and/or biological oceanographic variability as an explanation for the contrasting winter behavior between sexes. We aimed to confirm and explain the apparent differences in migration patterns by employing remotely sensed physical environmental fields and data collected by animal-borne sensors integrated into satellite transmitters. Based on the previous studies of NFS and other pinnipeds, we predicted that adult males and females would migrate to different regions of the North Pacific Ocean. We hypothesized that size dimorphism and local environmental conditions would explain intraspecific differences in diving and movement patterns and migration routes. We compared movement and diving parameters between the sexes to determine whether size predicts behavior and whether behavior is modulated by the habitat variables light (sun and moon), wind speed, and mixed-layer depth within and between ecosystems of the North Pacific Ocean and Bering Sea. As explained below, each of these variables has been shown to affect the movement and foraging behavior of fur seals and other pinnipeds and the geographic and diel vertical distribution of prey fields.

## Methods

### Ethics Statement

All work was conducted in accordance with and under the authority of the United States Marine Mammal Protection Act (National Marine Fisheries Service, NMFS Permits 782–1708 and 14328). The Marine Mammal Protection Act was established in 1972 requiring all research conducted on marine mammals in the United States be done under the authority of federal permits issued by either NMFS or U.S. Fish and Wildlife Service (USFWS). All applications for a permit to conduct research on marine mammals have gone through a four-stage review process that includes: 1) agency review (either NMFS or USFWS); 2) a public notice and review period; 3) review and recommendation from the Scientific Advisers to the U.S. Marine Mammal Commission; and 4) a final action by the reviewing agency. All capture and handling activities described in this manuscript have gone through and been approved by this process. Additionally, the University of Alaska Fairbanks Institutional Animal Care and Use Committee approved the capture and handling methods used on adult male NFS (Protocol 09–50). However, at the time when adult female NFS were captured by NMFS there was no additional requirement for review of these procedures by an institutional review board or ethics committee. In 2010, a NMFS Institutional Animal Care and Use Committee was established for the Alaska Fisheries and Northwest Fisheries science centers and the capture and handling protocols described here were reviewed and approved by this committee.

### Device Deployment

**Figure 1:**
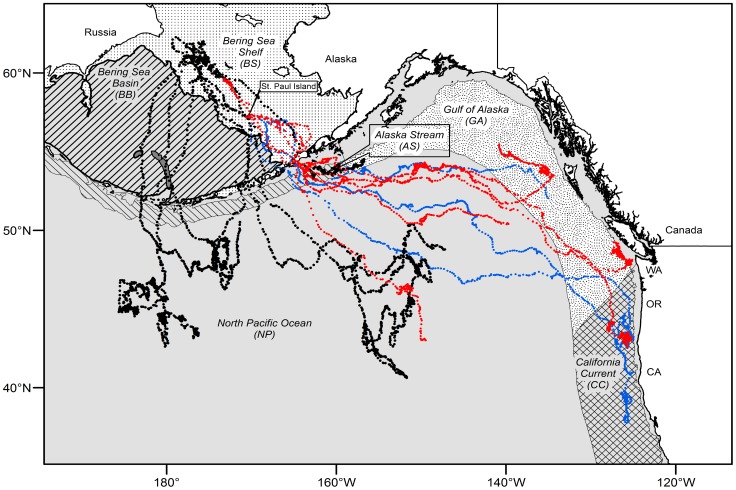
Adult male and female northern fur seal migratory routes. Map shows Large Marine Ecosystem delineations and all 6(the black dots), six adult females with diving data (the red dots), and four adult females without diving data (the blue dots).

We captured ten adult female and five adult male NFS on St. Paul Island (57.2° N, −172.2°W; Fig. 1) during October 2009 (Table 1). Females were captured, physically restrained, administered gas anesthesia (isoflurane and oxygen), weighed, and an Argos transmitting KiwiSat 202 satellite transmitter (Sirtrack LTD, Havelock North, New Zealand) or a “SPLASH” satellite-dive recorder (Wildlife Computers, Redmond, Washington, USA) was glued to the fur above the shoulder blades using 5-minute epoxy. Males were immobilized with an intramuscular injection (IM) using a jab stick or dart containing the drug combination Telazol (0.77–1.14 mg kg^−1^) and Medetomidine (0.03–0.04 mg kg^−1^). Isoflurane and oxygen were administered via mask in four males, and with an endotracheal intubation in one male, to ensure adequate procedural time to weigh and attach satellite-linked conductivity, temperature, depth satellite-relayed data loggers (SRDLs) developed by the Sea Mammal Research Unit at University of St. Andrews, Scotland. Following these procedures a drug reversal combination of Atipamezole (0.14–0.26 mg kg^−1^) and Flumazenil (0.002–0.005 mg kg^−1^) was given IM and each seal was monitored to complete recovery.

**Table 1:** Summary of capture information, satellite transmission durations, and Aleutian pass usage of adult male and female northern fur seals.

ID	Sex	Capture	Satellite	Dive	Mass	Capture	Migration	Last day of	Days post	Total	Aleutian passes
		site on	transmitter	data		date	departure	transmission	capture to	migrating	traveled through
		St. Paul Is.			(kg)		date		migration	days	
662	F	Polovina Cliffs	SPLASH	x	33.6	2-Oct-09	11-Nov-09	13-Mar-10	40.5	122.0	Unimak Pass
665	F	Polovina Cliffs	KiwiSat 202		37.8	5-Oct-09	23-Nov-09	23-Jan-10	49.5	60.5	Unimak Pass
670	F	Polovina Cliffs	SPLASH	x	35.6	2-Oct-09	5-Nov-09	23-Feb-10	34.5	110.0	Unimak Pass
671	F	Polovina Cliffs	SPLASH	x	44.0	3-Oct-09	12-Nov-09	22-Dec-09	40.3	40.0	Unimak Pass
672	F	Polovina Cliffs	SPLASH	x	28.4	3-Oct-09	14-Nov-09	28-Dec-09	42.0	44.3	Unimak Pass
673	F	Polovina Cliffs	SPLASH	x	32.4	3-Oct-09	30-Oct-09	12-Dec-09	27.8	42.5	Unimak Pass
674	F	Polovina Cliffs	SPLASH	x	45.4	3-Oct-09	13-Nov-09	4-Mar-10	41.5	110.5	Unimak Pass
675	F	Polovina Cliffs	KiwiSat 202		27.0	5-Oct-09	14-Nov-09	15-Nov-09	40.0	1.0	n/a
676	F	Polovina Cliffs	KiwiSat 202		38.0	5-Oct-09	12-Nov-09	11-Mar-10	38.0	119.0	Akutan Pass
677	F	Polovina Cliffs	KiwiSat 202		37.6	7-Oct-09	15-Oct-09	3-Mar-10	8.0	139.0	Unimak Pass
678	M	Zapadni Sands	SRDL	x	149.1	22-Oct-09	25-Oct-09	31-Jan-10	3.5	98.0	Unimak Pass
679	M	Zapadni Sands	SRDL	x	152.9	22-Oct-09	8-Nov-09	22-May-10	17.5	194.9	Buldir – Kiska Pass,
											Amukta Pass,
											Tanaga Pass
680	M	Zapadni Sands	SRDL	x	114.2	23-Oct-09	25-Oct-09	9-Apr-10	2.8	166.0	Amchitka Pass
681	M	Reef	SRDL	x	118.7	23-Oct-09	6-Nov-09	25-Dec-09	14.3	49.0	Yunaska Is. Pass
682	M	Zapadni Sands	SRDL	x	221.8	24-Oct-09	11-Nov-09	19-May-10	18.5	189.4	Samalga Pass

F = female; M = male.

All three satellite-transmitter types provided daily location estimates, while the two types of dive recorders (SPLASH and SRDL) stored and transmitted summary dive information. SPLASH satellite-dive recorders sampled depth every second and stored all dives ≥2 m (dive depth and duration) in 6 h blocks as histogram distributions starting at 0000, 0600, 1200, 1800 UTC [12]. Fourteen histogram dive depth bins (2, 4, 6, 10, 20, 34, 50, 74, 100, 124, 150, 174, 200, >200 m) and dive duration bins (15, 30, 60, 90, 120, 150, 180, 210, 240, 300, 330, 360, >360 s) were defined prior to tag deployment. Each dive depth represented the maximum depth recorded during a dive, while each dive duration represented the total time of the entire dive, beginning and ending at 2 m depth. Maximum dive depth and duration were recorded and then stored in the corresponding predefined histogram bin. SRDLs sampled depth every second and recorded dives that were >6 m for at least 8 seconds. Like the SPLASH recorders, dive summaries were stored every 6 h at 0000, 0600, 1200, and 1800 UTC. However, unlike SPLASH recorders, SRDLs computed onboard and transmitted the average and standard deviation of dive depths, maximum of the dive depths, average and standard deviation of the dive durations, maximum of the dive durations, and the total number of dives for each 6 h time block.

### Dive Data Processing and Analyses

#### Satellite-Relayed data loggers (Males)

Satellite-relayed data loggers provided several measures of dive and environmental characteristics. We used average dive depth and dive duration, maximum dive depth and dive duration, and the number of dives for each 6 h block, as well as all conductivity, temperature, and depth (CTD) profiles. The CTD profiles were calculated from the deepest dive in each 6 h block as long as the dive exceeded ∼4 m. Once the deepest dive was detected, the SRDL switched into sampling mode on the ascent portion of the dive by sampling temperature, conductivity, and depth at 1 Hz until the seal reached the surfaced. Several quality control measures were applied to the profile before 17 representative depth points (8 predefined, 7 inflection, minimum, and maximum) were selected for transmission [34]. Our goal for the CTD data was to determine the mixed-layer depth (MLD) for each 6 h block. To do so, we employed the algorithm described by Kara et al. [35], which uses a seasonally-optimized density difference criterion as a practical definition of the thickness of the well-mixed layer near the ocean surface. We chose this algorithm because it was calibrated using climatological data obtained from CTD casts along Canada’s Department of Fisheries and Oceans “Line P”, in the northeast Pacific, in a region proximate to the winter foraging grounds of NFS [36], [37]. Starting from just below the surface, the Kara et al. [35] algorithm scans successively deeper samples from a given cast to find the vertical extent of the surface mixed layer of uniform density (σ_t_). The MLD was then defined as the depth at which density rose by a specified increment (Δσ_t_) above the density at the deepest sample in the mixed layer.

To identify the mixed layer for a given dive, the algorithm searched from 10 m for the first observed depth interval between samples *n* and *n+*1 (described as depth *h_n_* to depth *h_n+1_*) in which the density increase between the two samples was greater than 10% of Δσ_t_. The density increment Δσ_t_ was defined as the density increase that would result from reducing the temperature at *h_n_* by the seasonally-prescribed value ΔT. In keeping with the values found by Kara et al. [35] to produce the best results along Line P, we used ΔT = 1.0°C in winter (January-March), ΔT = 0.8°C in spring (April-June), ΔT = 0.2°C in summer (July-September), and ΔT = 0.8°C in fall (October-December). Once a pair of samples meeting the above criteria was found, the density at *h*_n_ was then defined as the reference density (σ_t, ref_) and the depth at which observed density exceeded σ_t, ref_+Δσ_t_ was the MLD.

Additionally, we employed various quality control measures to eliminate dives with erroneous density spikes or other outliers. Since the Kara et al. [35] algorithm assumes a minimum MLD of 10 m, for dives with CTD measurements whose maximum depth did not exceed 10 m, it was assumed that the maximum dive depth was shallower than the MLD. For dives that detected large density decreases or unstable regions in the top of the water column we used a secondary method in which the 10 m density was used as a reference rather than the well-mixed layer base. For dives with weak density stratification, where the algorithm could not find a MLD from the sample-pair method by the depth at which density increases by 2Δσ_t_ from the 10 m reference value, it again defaulted to the secondary method. If neither method was successful, it was presumed the maximum depth for that dive was shallower than the MLD.

#### SPLASH (Females)

Dive data were processed using Wildlife Computers’ WC-DAP program (V.2.0.29) and then filtered, quality controlled for spurious outliers, and analyzed using methods similar to those outlined by Lea et al. [12]. Duplicate histogram records were removed, only dives >6 m were analyzed in order to be consistent with male SRDL dive depth summaries, and dive durations >15 s were selected to reduce the effect of non-foraging behavior. The average dive depth and average dive duration were computed using each dive depth and dive duration histogram distribution and applying the same formula and methods described in Lea et al. [12].

### Modeling Movement

Satellite tag transmission intervals were set to receive multiple daily location estimates [38]. The SRDLs were programmed to transmit all hours between noon and 10∶00 UTC the next day; SPLASH tags were set to transmit between 0200–0600 and 1400–1800 UTC; and all four KiwiSat 202 s were set to transmit between 0030–0600, with one tag additionally transmitting between 1400–1500 and another between 1900–2350 UTC. Within these transmission intervals, Argos location estimates occur at irregularly spaced time intervals with some observation error. Our study objectives were to combine seal behavioral response variables, both horizontal movement and dive behavior, with remotely sensed fields and animal-borne CTD measurements. Seal dive behavior, animal-borne CTD measurements, and derived wind speeds and directions all summarized measurements taken in 6 h time blocks (see below). Thus, we needed to estimate locations every 6 h from the Argos location data set and quantify the seal movement behavior. To do this, we fitted a switching state-space model (SSSM) for each seal to account for the Argos observation error, create animal location estimates every 6 h, and identify movement behavior as either resident (area restricted or foraging movements) or transient (fast, somewhat linear or directed movements) [39]–[43]. Switching state space models were estimated with Markov Chain Monte Carlo estimation methods using WinBUGS and implemented through R statistical software and the R2WinBUGS package (http://www.r-project.org).

### Predictors of Movement and Diving Behavior

Our hypothesis explaining adult male and female spatial segregation during their winter migration includes the confluence of NFS biology–the evolutionary effects of the mating system on sexual dimorphism [11]–and behavioral and environmental effects on NFS prey distribution. Specifically, we predicted deeper and longer dives for animals with greater mass, allowing the larger males to forage in regions where biophysical processes and behavioral adaptations deepen prey fields. Factors known to affect NFS potential prey include light (both sun and moon), season, and habitat [12], [16],[28],[44], and to lesser extents the depth of the surface mixed layer [8] and winter storms [13]. Spatial and temporal alignment between NFS behavior (movement and diving) and light (sun and moon), wind speed and direction, surface MLD, ecosystem (defined and discussed as Large Marine Ecosystems, LMEs, see below), and season was accomplished by the following methods.

#### Proportion daylight

The proportion of daylight in each 6 h period was calculated using custom coded functions in R that utilized the National Oceanic and Atmospheric Administration Sunrise/Sunset and Solar Position Calculators (http://www.srrb.noaa.gov/highlights/sunrise/sunrise.html). Nautical dawn and dusk (solar elevation equal to 12° below horizon) were calculated using each seal’s estimated latitude and longitude at the midpoint of the 6 h block, and proportion daylight was defined as the amount of the 6 h block within daylight hours.

#### Lunar fraction

The lunar fraction is the illuminated area of the moon’s apparent disk divided by the total area of the disk. Calculations of the lunar fractions were extracted from the United States Naval Observatory website (http://aa.usno.navy.mil/data/docs/MoonFraction.php) and temporally aligned with all 6 h periods containing summaries of dive behavior.

#### Wind speed and direction

National Centers for Environmental Prediction and National Center for Atmospheric Research (NCEP-NCAR) Reanalysis 2 data distributed by the NOAA/OAR/ESRL PSD, Boulder, Colorado, USA (http://www.esrl.noaa.gov/psd/) provided sub-daily analysis estimates of 10 m *u* (east-west) and *v* (north-south) wind velocities at 0000, 0600, 1200, and 1800 UTC on a 2.5° latitude × 2.5° longitude global grid. Seal location estimates from the SSSM were estimated at the same sub-daily time intervals and spatially and temporally aligned with *u* and *v* velocities. The *u* and *v* velocities were then converted to wind speed (m s^−1^) and wind direction. The validity of atmospheric reanalyses for characterizing the winds of the North Pacific has been previously assessed. Using wind data from moored buoys that were not available for assimilation in the NCEP-NCAR Reanalysis, [45] found complex correlation coefficients between the measured and synthetic winds of about 0.9, with minimal systematic biases.

#### Mixed-Layer depth (Males)

Mixed-layer depth calculations (see SRDL above) were temporally aligned with each seal location estimate. If more than one CTD profile and subsequent MLD calculation temporally corresponded to a single 6 h dive summary, then the average MLD was computed. Seventy-three percent of the 6 h dive summary bins had a corresponding MLD calculation. For some of the 6 h dive summaries without MLD calculations it was appropriate to estimate its depth by linear interpolation, particularly if the time gap was small and/or the variability in the MLD was low prior to and after the missing MLD calculations. This was a subjective process that increased paired MLD calculations and 6 h dive summaries to 86% of the total number of 6 h dive summaries.

#### Oceanic habitats

During their seasonal migration, NFS traverse marine ecosystems characterized by distinct hydrographic and topographic domains, productivity, and upper trophic level assemblages [16], [28], [30], [46]. We determined the physical extent and boundaries of these marine ecosystems by modifying LMEs of the World map (http://www.lme.noaa.gov) [47]. Modifications incorporated continental shelf (≤1000 m) and oceanic ecosystems, including the addition of an Alaska Stream ecosystem, adjusting the East Bering Sea LME to lie north of the Aleutian Islands while further distinguishing Bering Sea shelf (≤1000 m) and basin (>1000 m) ecosystems, and adding the North Pacific Ocean ecosystem (Fig. 1). The LMEs we used in the statistical analyses were the Bering Sea Shelf (BS), Bering Sea Basin (BB), Alaska Stream (AS), North Pacific Ocean (NP), Gulf of Alaska (GA), and California Current (CC). References to the Bering Sea refer to both the BB and BS ecosystems.

#### Season

Days since 1 October (referred to as season) were used to estimate any temporal signal in the data for each 6 h estimated location.

### Statistical Analyses

#### Mass and dive behavior

Linear models were used to investigate the relationship between NFS mass and dive behavior. Mass was used as a predictor variable for both male and female average dive depths and durations, as well as the average maximum dive depths and durations. In addition, we investigated the relationship between the average numbers of dives with respect to male and female average dive depths.

#### Movement and dive behavior

Biophysical processes responsible for shaping the distribution, depth, and density of NFS preferred prey fields may also influence the horizontal movement and vertical diving behavior of individuals. Northern fur seal movement and diving behavior were simplified into three response variables – SSSM estimated movement behavioral state (hereinafter referred to as behavioral state), average dive depth every 6 h, and the total number of dives every 6 h. The SSSM provided an index of movement behavior scaled continuously between 0 and 1, with 0 indicating transient or fast moving linear movements and 1 indicating area-restricted resident or foraging movements [41]. To assist with normality we added 0.0001 to values equal to 0 and subtracted 0.0001 from values equal to 1 prior to logit transforming all SSSM behavioral state values [40]. Both the average dive depth and total number of dives every 6 h were log transformed.

Linear mixed-effects models were used to assess the relationship between movement and dive behavior with respect to light (sun and moon), wind speed, ecosystem, season, sex, and mixed-layer depth (adult males only). Models were built and computed by using the nlme V3.1-103 package [48] in the R statistical software package V2.14.1. In all models, individual seal was used as a random effect, an AR(1) autocorrelation structure within each seal was assumed, and several model combinations of the main effects and interaction terms were contrasted, ranked, and selected using Akaike’s Information Criterion. Specifically, the response variable SSSM behavioral state was assessed with respect to wind speed, sex, season, and ecosystem. Linear mixed-effects models used for examining seal dive behavior were built separately for adult males and females due to the addition of the mixed-layer depth predictor variable to adult male diving response variables. We assessed the relationship between the average dive depth and number of dives in each 6 h period with respect to lunar fraction, proportion daylight in each 6 h dive period, ecosystem, season, and the mixed-layer depth (males only).

### Qualitative Assessment of Winter Winds and Mesoscale Variability

Finally, changes in North Pacific Ocean wind patterns–specifically the increase in storm frequency and intensity [49],[50] coupled with mesoscale oceanographic variability [51]–motivated a broad-scale seasonal qualitative assessment of spatial variation of high wind speeds and NFS eddy interactions. While we did not directly address the question of how the prevailing high wind patterns affected NFS population distribution as a whole, we discuss possible mechanisms to explain the distribution of adult males and females during the 2009/2010 winter and how high winds, mesoscale physical oceanographic variability, and NFS behavior are intertwined. For this, we employed two data sets to evaluate spatial wind speed and oceanographic variability over the entire NFS foraging range. NCEP-NCAR Reanalysis 2 10 m wind data were assessed for November through March by computing the proportion of days that wind speeds exceeded 11 m s^−1^ for each 2.5° latitude × 2.5° longitude grid cell–these were categorized as stormy days or high winds. For oceanographic variability we aligned fur seal locations with the weekly distribution of the eddy field as determined by Chelton et al. [51] and available online from http://cioss.coas.oregonstate.edu/eddies/. This dataset covers the time period October 1992 to January 2011 and utilizes Version 3 of the AVISO Reference Series gridded sea surface height product to identify eddy center latitudes and longitudes, eddy radius scale, rotation direction, and approximate eddy strengths at 7-day time steps. At each 7-day interval we calculated the distance between seals to the closest eddy edge by using multi-dimensional visualization software (Eonfusion, Myriax Pty Ltd, Hobart, Australia).

## Results

**Figure 2:**
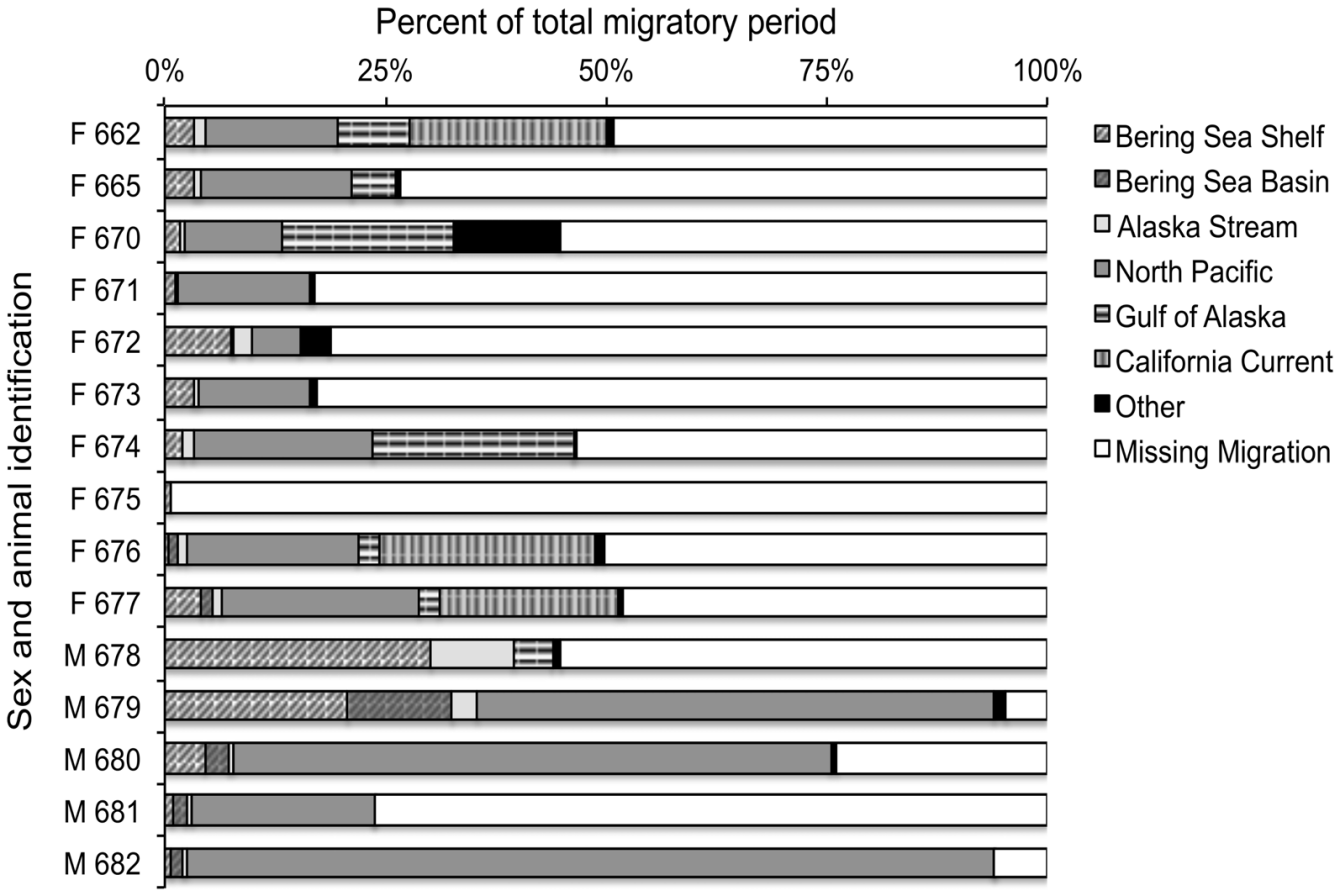
Percent of the estimated total adult male and female northern fur seal migratory period spent in each ecosystem. To make this calculation, we assumed return dates of 1 June for males (M) and 10 July for females (F).

All 15 satellite transmitters communicated for >40 d after deployment. One adult female’s transmitter stopped working one day into her winter migration after recording 40 days of foraging trips (not used in analyses) to the north of St. Paul Island, and transmitters of three females ceased working early into their winter migration while transiting the NP. We estimated the total migration duration by assuming a return date to St. Paul Island of 1 June for males and 10 July for females [11], [52]. Using these return dates, we estimated that 45–52% of the adult female migration was recorded for half of the females tagged (Fig. 2); the longest migratory tracking period was 139 d (Table 1). Three of the five male SRDL tags transmitted into the spring months, recording an estimated 76–95% of the total migratory period, with the longest tracking duration lasting 195 d. The other two male tags stopped transmitting after 49 d and 98 d into the migration (Table 1, Fig. 2).

### Initial Dispersal

Initial dispersal from St. Paul Island occurred between 25 October and 23 November, with 10 seals departing within a 10 d period associated with high wind speeds over the island generated by two storms (mean wind speed = 12 m s^−1^, maximum 6 h average = 22 m s^−1^). Two females remained in the Bering Sea for 14 d and 18 d before exiting into the NP, while the other seven females traveled out of the Bering Sea in less than 8 d. One female traveled through Akutan Pass and the remaining eight females traveled through nearby Unimak Pass (Table 1 and Fig. 1).

Two males traveled directly south from St. Paul Island and exited the Bering Sea in less than 4 d. The other three spent 16 d, 23 d, and 66 d foraging in the Bering Sea prior to traveling south and into the NP. Only one male traveled through Unimak Pass, while the others exited through passes further to the west (Table 1 and Fig. 1). One male traveled through the Aleutian Islands on three occasions in different locations (between Buldir and Kiska islands, Amukta Pass, and Tanaga Pass) as a result of foraging alternately in the NP and Bering Sea.

### Ecosystem Preference

Destination areas and ultimate overwintering ecosystems varied by sex (Figs. 1 and 2). Adult females transited large regions of the NP to forage in the GA and CC. Of the four females whose tags stopped transmitting early, one ceased working while in the BS, one ceased working shortly after entering the NP, one appeared to be heading south toward the Transition Zone Chlorophyll Front (TZCF) [15], [16], and one was heading toward the GA. The remaining six females averaged 43±11.5 SD days to transit the NP before arriving in the GA or CC. Four of the five males remained in the NP and Bering Sea and did not enter the GA or CC; the other male, with the shortest transmission duration, exited the BS and foraged in the AS before contact was lost.

### Effects on Behavioral State

**Figure 3:**
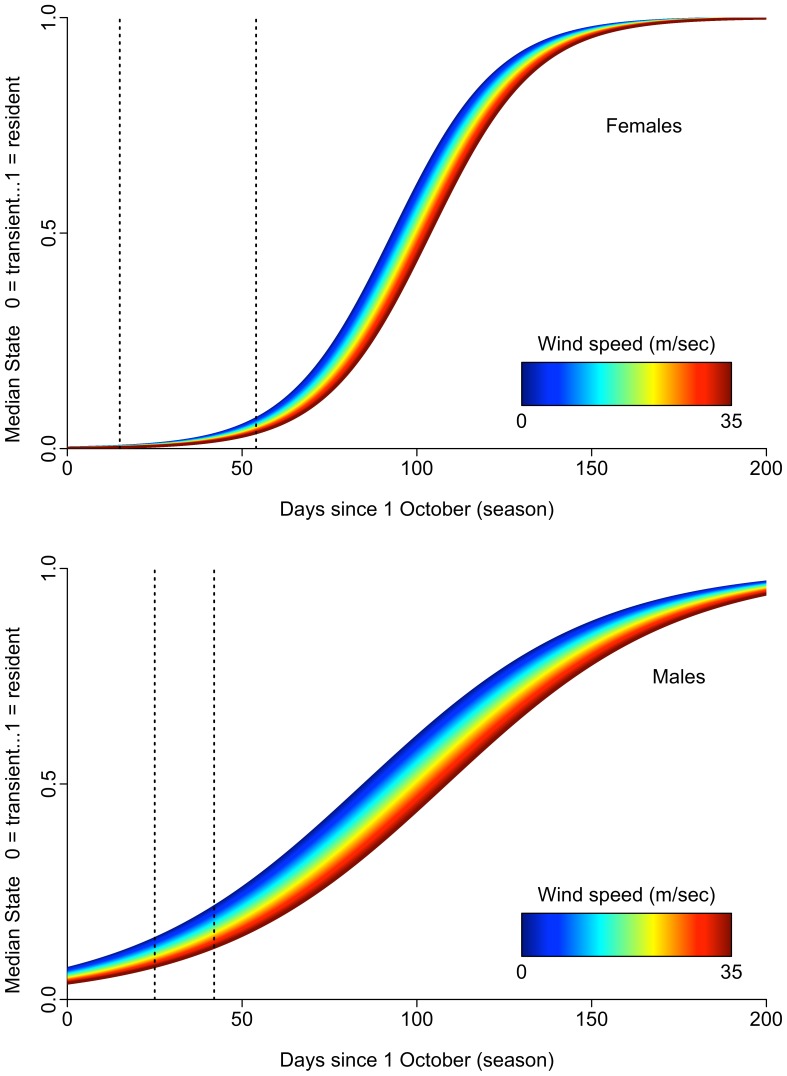
The effect of wind and season on adult male and female northern fur seal behavioral states. These figures were constructed using the linear mixed-effects model coefficients from Table 3 depicting days since 1 October (season) and wind speed. The vertical dashed lines indicate the first and last migration departure date of the seals in this study.

Of the top three linear mixed-effects models examining adult male and female behavioral state (Table 2), the best model identified season, wind speed, ecosystem, sex, and the interaction between season and sex as important predictors (Table 3). Model results revealed that high wind speeds led to a more transient behavioral state (lower index), and as the season progressed both males and females moved toward a resident behavioral state (higher index). The slope of this seasonal progression differed between the sexes due to females reaching a resident behavioral state earlier in the season compared to males (Table 3, Fig. 3). In addition, the extremely low behavioral states (indicating fast linear movements) of females within the first two months of their migration are consistent with previous observations of adult females rapidly exiting the BS and transiting the AS and NP en route to the GA and CC ecosystems.

**Table 2:** Top three linear mixed-effects models selected using Akaike Information Criterion (AIC) for each adult male and female northern fur seal response variable examined.

GLMM model	K	AIC	ΔAIC
(1) Behavioral state (*n = *5946)			
(a) WS+sex+S+LME (NP, AS, BB, BS, CC, GA)+sex*S	13	16449.15	
(b) WS+sex+S+LME (NP, AS, BB, BS, CC, GA) + S*LME (NP, AS, BB, BS, CC, GA)	17	16451.39	2.24
(c) WS+sex+S+sex*S	8	16451.88	2.73
(2) F average dive depth (*n* = 893)			
(a) D+M+LME (NP, AS, BS, CC, GA)+D*M+LME (NP, AS, BS, CC, GA)*M + LME (NP, AS, BS, CC, GA)*D	19	1496.00	
(b) D+M+LME (NP, AS, BS, CC, GA)+D*M+LME (NP, AS, BS, CC, GA)*M	15	1498.30	2.30
(c) D+M+S+LME (NP, AS, BS, CC, GA)+D*M	16	1500.07	4.07
(3) M average dive depth (*n* = 1473)			
(a) D+M +S+LME (NP, AS, BB, BS)+D*MLD+M*D+M*LME (NP, AS, BB, BS)	15	2508.14	
(b) D+S+LME (NP, AS, BB, BS)+D*MLD+M*LME (NP, AS, BB, BS)	14	2508.63	0.49
(c) D+M+LME (NP, AS, BB, BS)+D*MLD+M*D+M*LME (NP, AS, BB, BS)	14	2510.79	2.65
(4) F number of dives (*n* = 893)			
(a) D+LME (NP, AS, BS, CC, GA)+LME (NP, AS, BS, CC, GA)*D	13	2862.60	
(b) D+LME (NP, AS, BS, CC, GA)+LME (NP, AS, BS, CC, GA)*D+D*M	14	2864.58	1.98
(c) D+M+LME (NP, AS, BS, CC, GA)+LME (NP, AS, BS, CC, GA)*D+D*M	15	2866.48	3.88
(5) M number of dives (*n* = 1473)			
(a) D+LME (NP, AS, BB, BS)+LME (NP, AS, BB, BS)*D+D*MLD	12	4299.75	
(b) D+M+LME (NP, AS, BB, BS)+LME (NP, AS, BB, BS)*D+D*MLD	13	4301.13	1.38
(c) D+M+LME (NP, AS, BB, BS)+LME (NP, AS, BB, BS)*D+D*MLD+D*M	14	4303.12	3.37

F = female; M = male; K = number of parameters; NP = North Pacific Ocean; AS = Alaska Stream; BB = Bering Sea Basin; BS = Bering Sea Shelf; CC = California Current; GA = Gulf of Alaska; WS = wind speed (m sec^−1^); S = season or days from 1 October; D = proportion of daylight in each 6 h period; M = fraction of the moon illuminated; MLD = mixed-layer depth.

**Table 3:** Best linear mixed-effects model results and estimated coefficients for the effects on adult male and female northern fur seal behavioral state.

Behavioral state	Estimate	*SE*	*df*	*t*	*P*
Intercept	−6.15	1.09	5923	−5.63	**<0.0001**
WS	−0.02	0.00	5923	−5.88	**<0.0001**
sex (male)	3.60	1.46	13	2.47	**0.0284**
S	0.07	0.01	5923	5.47	**<0.0001**
AS	−0.71	0.23	5923	−3.07	**0.0021**
BB	−0.74	0.39	5923	−1.91	0.0561
BS	−0.91	0.34	5923	−2.67	**0.0075**
CC	−0.76	0.59	5923	−1.29	0.1966
GA	−0.41	0.28	5923	−1.45	0.1463
sex*S	−0.04	0.01	5923	−3.04	**0.0024**

The reference levels are females and North Pacific Ocean. *P*-values in boldface are significant at *P*≤0.05. AS = Alaska Stream; BB = Bering Sea Basin; BS = Bering Sea Shelf; CC = California Current; GA = Gulf of Alaska; WS = wind speed (m sec^−1^); S = season or days from 1 October.

### Mass Effects on Adult Male and Female Dive Depths and Dive Durations

**Figure 4:**
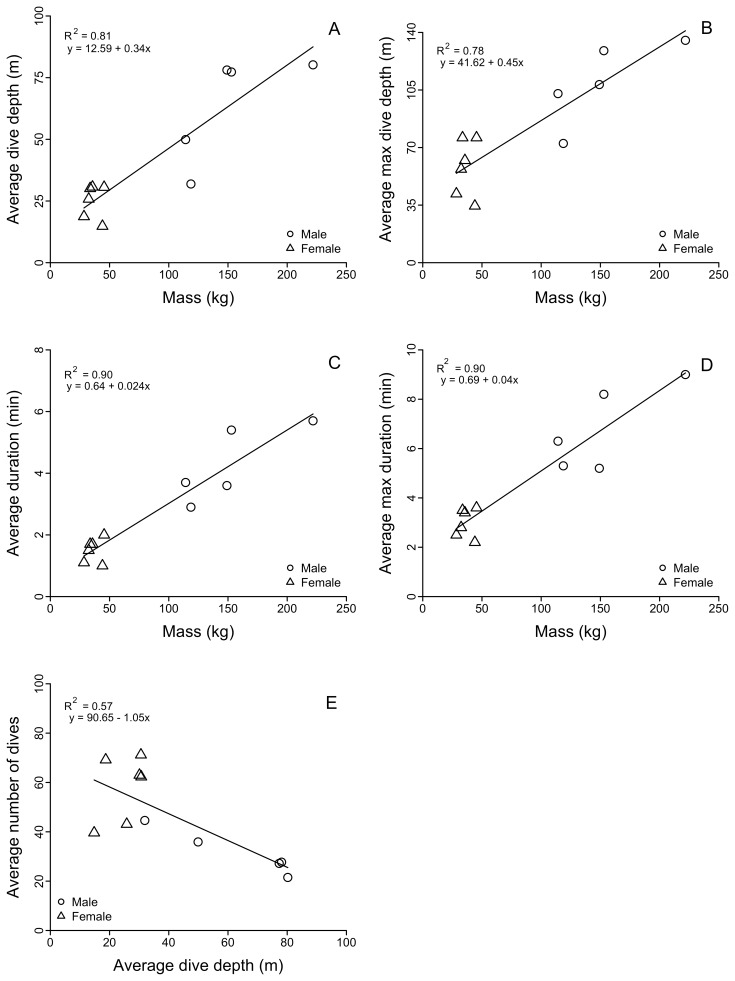
Relationships between adult male and female northern fur seal mass and dive behaviors. Significant linear regression relationships were found between fur seal mass and their average dive depth (A), average maximum dive depth (B), average dive duration (C), and average maximum dive duration (D). The relationship between average fur seal dive depths and the average number of dives (E) was also significant.

Adult males dove deeper and for longer durations than adult females, and average dive depth (Table 4, Fig. 4A; R^2^ = 0.81, df = 9, *P*<0.001), average maximum dive depth (Fig. 4B; R^2^ = 0.78, df = 9, *P*<0.001), average dive duration (Fig. 4C; R^2^ = 0.90, df = 9, *P*<0.001), and average maximum dive duration (Fig. 4D; R^2^ = 0.90, df = 9, *P*<0.001) were all positively correlated with seal mass. However, larger, deeper diving males performed fewer dives per 6 h compared to their female counterparts (Fig. 4E; R^2^ = 0.57, df = 9, *P* = 0.007).

**Table 4:** Average dive depth and the percentage of dives of adult male and female northern fur seals summarized for each Large Marine Ecosystem (LME) and grouped by daylight periods.

		Mean dive depth in meters ± SD				Percentage of dives (number of dives)								
LME	sex	day	>50% day	>50% night	night	day		>50% day		>50% night		night		Total Dives
BB	F													0
	M	117.4±54.0	115.7±24.1		39.6±40.8	62%	(966)	5%	(84)	0%	(0)	32%	(503)	1,553
BS	F	42.4±20.6	24.6±15.8		27.1±23.4	9%	(205)	17%	(378)	0%	(8)	74%	(1,679)	2,270
	M	78±45.0	74.8±50.0		62.4±36.7	19%	(1,240)	9%	(583)	0%	(0)	73%	(4,874)	6,697
NP	F	40.3±26.0	27.5±22.2	22.1±17.3	23.5±23.0	2%	(584)	18%	(4,749)	32%	(8,254)	47%	(12,101)	25,688
	M	90±40.2	64.6±32.6	46.7±30.7	24.8±18.3	63%	(24,436)	12%	(4,781)	13%	(5,179)	12%	(4,696)	39,092
AS	F	21.1±16.4	18.9±8.5		15.6±6.6	4%	(170)	17%	(697)	0%	(0)	79%	(3,205)	4,072
	M	158.7±72.0	169.3±58.2		31.9±33.7	51%	(711)	32%	(450)	0%	(0)	17%	(231)	1,392
CC	F	37.1±19.5	28.5±13.1	25.3±13.4	22.7±10.6	33%	(1,323)	46%	(1,857)	10%	(391)	11%	(437)	4,008
	M													0
GA	F	33.1±41.8	31.4±15.4	39.6±22.9	29.7±22.5	0%	(31)	17%	(3,115)	41%	(7,460)	42%	(7,559)	18,165
	M													0

NP = North Pacific Ocean; AS = Alaska Stream; BB = Bering Sea Basin; BS = Bering Sea Shelf; CC = California Current; GA = Gulf of Alaska; F = female; M = male.

### Exogenous Effects on Dive Depth

**Figure 5:**
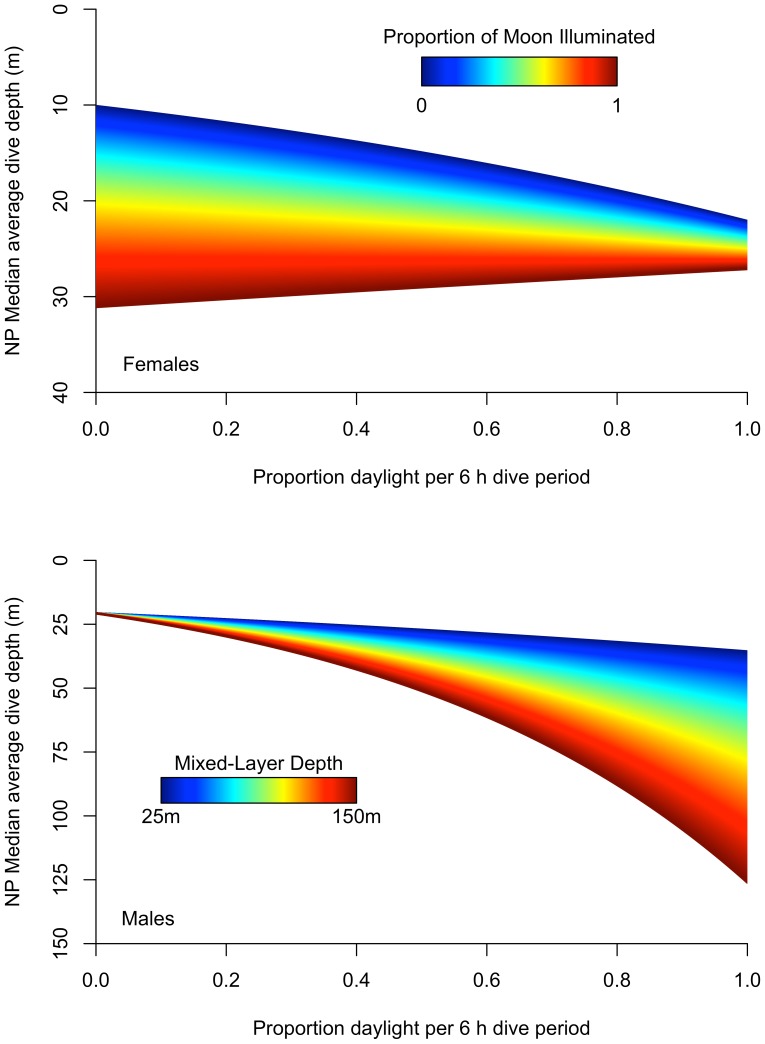
Dive response of adult male and female northern fur seals to light (moon and sun) and mixed-layer depth. These figures were constructed using the linear mixed-effects model coefficients from Table 5. The top figure shows the effect of moonlight and sunlight on female dive behavior while the bottom figure shows the effect of sunlight and mixed-layer depth on male dive behavior. Both plots represent adult diving in the North Pacific Ocean (NP) ecosystem.

Of the top three linear mixed-effects models examining adult female dive depths (Table 2), the best model identified daylight, moonlight, and variation between ecosystems and interactions between daylight, moonlight, and ecosystem as explanatory variables (Table 5). As the percentage of daylight in each 6 h period and the lunar fraction increased, female dive depths increased in the NP (Fig. 5 top). When contrasted to the NP, high lunar fraction in the BS, AS, and GA ecosystems had less of an effect on female dive depths, and dives which took place during the night with a new moon resulted in significantly deeper dives (Table 5). Adult males showed a similar response to increased sunlight levels by also diving deeper during the day and to moonlight levels by diving deeper at night on moonlit nights, particularly in the BS (Tables 4 and 5). As expected, with increasing daylight the lunar effect decreased in females but not significantly for males, which may reflect the day-night differences in preferred foraging times between the sexes (Table 4, see next section). For males, as the percent daylight in each 6 h period increased, the MLD became a more significant influence on dive depths, leading to correspondence between their dive depths and MLD during the daytime (Table 5, Fig. 5 bottom). In addition, male dive depths responded to seasonal changes in the MLD by slightly increasing in depth in winter and shoaling in spring.

**Table 5:** Best linear mixed-effects model results and estimated coefficients for the effects on adult male and female northern fur seal average dive depths.

Female Dive Depth	Estimate	*SE*	*df*	*t*	*P*
Intercept	2.314	0.092	872	25.044	**<0.0001**
D	0.782	0.115	872	6.781	**<0.0001**
M	1.122	0.124	872	9.071	**<0.0001**
AS	0.298	0.147	872	2.025	**0.0432**
BS	0.611	0.159	872	3.843	**0.0001**
CC	0.158	0.208	872	0.758	0.4487
GA	0.711	0.111	872	6.396	**<0.0001**
D*M	−0.918	0.155	872	−5.929	**<0.0001**
M*AS	−0.903	0.321	872	−2.811	**0.0051**
M*BS	−0.995	0.260	872	−3.832	**0.0001**
M*CC	−0.502	0.264	872	−1.900	0.0578
M*GA	−0.882	0.167	872	−5.273	**<0.0001**
D*AS	−0.320	0.185	872	−1.727	0.0845
D*BS	0.161	0.170	872	0.947	0.3441
D*CC	0.357	0.189	872	1.891	0.0589
D*GA	0.071	0.136	872	0.523	0.6009
**Male Dive Depth**	**Estimate**	***SE***	***df***	***t***	***P***
Intercept	3.031	0.156	1457	19.424	**<0.0001**
D	0.290	0.110	1457	2.635	**0.0085**
M	0.218	0.092	1457	2.369	**0.0179**
S	−0.001	0.001	1457	−2.158	**0.0311**
AS	1.374	0.182	1457	7.553	**<0.0001**
BB	0.167	0.154	1457	1.084	0.2785
BS	0.353	0.112	1457	3.149	**0.0017**
M*AS	−0.657	0.276	1457	−2.379	**0.0175**
M*BB	0.196	0.259	1457	0.759	0.4479
M*BS	0.616	0.152	1457	4.050	**0.0001**
D*M	−0.155	0.099	1457	−1.564	0.1180
D*MLD	0.010	0.001	1457	10.440	**<0.0001**

Females and males were examined separately and the reference level is the North Pacific Ocean. *P*-values in boldface are significant at *P*≤0.05. AS = Alaska Stream; BB = Bering Sea Basin; BS = Bering Sea Shelf; CC = California Current; GA = Gulf of Alaska; S = season or days from 1 October; D = proportion of daylight in each 6 h period; M = fraction of the moon illuminated; MLD = mixed-layer depth.

### Exogenous Effects on Dive Activity

**Figure 6:**
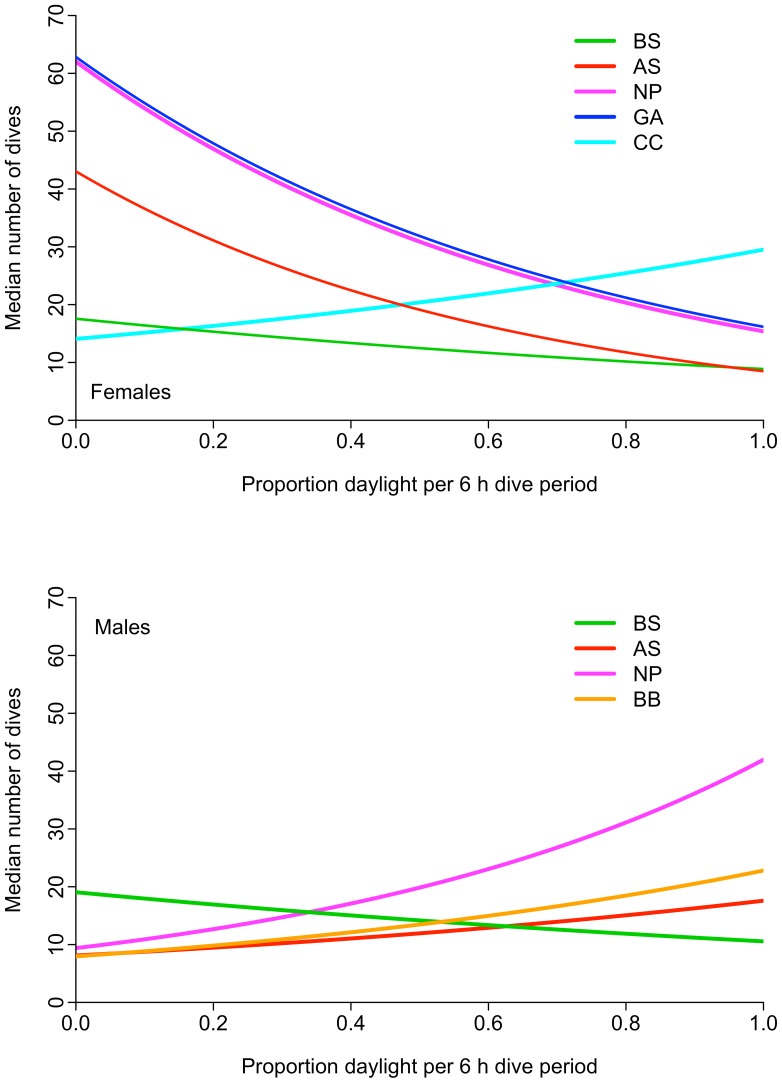
The effect of daylight on the number of dives of adult male and female northern fur seals. Each line represents an ecosystem: NP = North Pacific Ocean, AS = Alaska Stream, BB = Bering Sea Basin, BS = Bering Sea Shelf, CC = California Current, GA = Gulf of Alaska (see Fig. 1). In most cases, male dives (bottom) increased with increased proportion of light in each 6 h period. This response was opposite of adult females (top), which dived more during the night; a notable exception occurred in the CC ecosystem where one female exhibited an increase in the number of dives per 6 h period with increasing daylight.

Of the top three linear mixed-effects models examining number of dives per 6 h period in adult females and males (Table 2), the best model showed that females had a greater number of dives per 6 h during the night in four of five LMEs visited–the exception was the CC where the only female with a functioning dive recorder performed more dives in each 6 h period with increasing proportion of daylight (Tables 4 and 6, Fig. 6 top). These trends are consistent with those of females tagged in previous years with dive recorders (unpublished data) [16], and thus are not likely an artifact of the small sample size in this study but instead represent ecosystem dive response. In contrast to adult females foraging in the AS, NP, and GA, adult males increased the number of dives with an increased proportion of daylight in each 6 h period while foraging in the AS, NP, and BB (Table 6, Fig. 6 bottom). The exception for male seals was the BS, a neritic region limiting the depth of prey to 200 m or less. Unlike their dive behavior in pelagic ecosystems (NP, BB, and AS), males performed more dives at night (73%) in the BS, a similar dive pattern as adult females foraging in the BS (Table 4).

**Table 6:** Best linear mixed-effects model results and estimated coefficients for the effects on adult male and female northern fur seal average number of dives.

Female number of dives	Estimate	*SE*	*df*	*t*	*P*
Intercept	4.128	0.121	878	34.057	**<0.0001**
D	−1.394	0.211	878	−6.613	**<0.0001**
AS	−0.365	0.225	878	−1.621	0.1054
BS	−1.262	0.231	878	−5.457	**<0.0001**
CC	−1.483	0.297	878	−4.998	**<0.0001**
GA	0.013	0.155	878	0.084	0.9333
D*AS	−0.230	0.413	878	−0.556	0.5783
D*BS	0.710	0.389	878	1.826	0.0683
D*CC	2.135	0.432	878	4.939	**<0.0001**
D*GA	0.036	0.323	878	0.113	0.9104
**Male number of dives**	**Estimate**	***SE***	***df***	***t***	***P***
Intercept	2.248	0.202	1460	11.152	**<0.0001**
D	1.498	0.204	1460	7.340	**<0.0001**
AS	−0.147	0.298	1460	−0.493	0.6222
BB	−0.173	0.198	1460	−0.874	0.3823
BS	0.700	0.125	1460	5.612	**<0.0001**
D*AS	−0.724	0.372	1460	−1.949	0.0515
D*BB	−0.446	0.273	1460	−1.630	0.1034
D*BS	−2.088	0.181	1460	−11.527	**<0.0001**
D*MLD	0.000	0.002	1460	−0.076	0.9393

Females and males were examined separately and the reference level is the North Pacific Ocean. *P*-values in boldface are significant at *P*≤0.05. AS = Alaska Stream; BB = Bering Sea Basin; BS = Bering Sea Shelf; CC = California Current; GA = Gulf of Alaska; D = proportion of daylight in each 6 h period; MLD = mixed-layer depth.

### Winter Winds and Mesoscale Eddies

**Figure 7:**
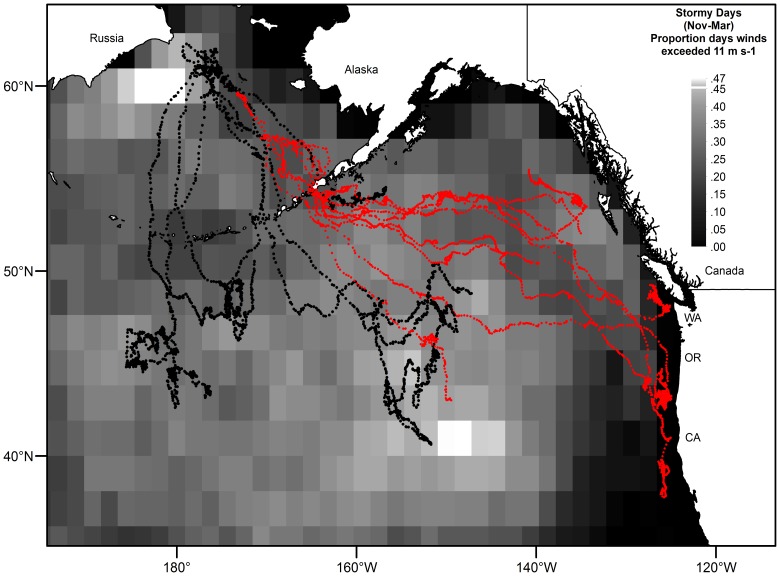
Spatial distribution of stormy days in relation to adult male and female northern fur seal migratory tracks. Stormy days, defined as the proportion of days wind speeds were greater than 11^−1^ [53] during the November-March time period, were compared to male (the black dots) and female (the red dots) migratory tracks. Males remained in the stormy regions of the North Pacific Ocean, while females traveled to regions experiencing fewer stormy days along the continental margins of North America.

**Figure 8:**
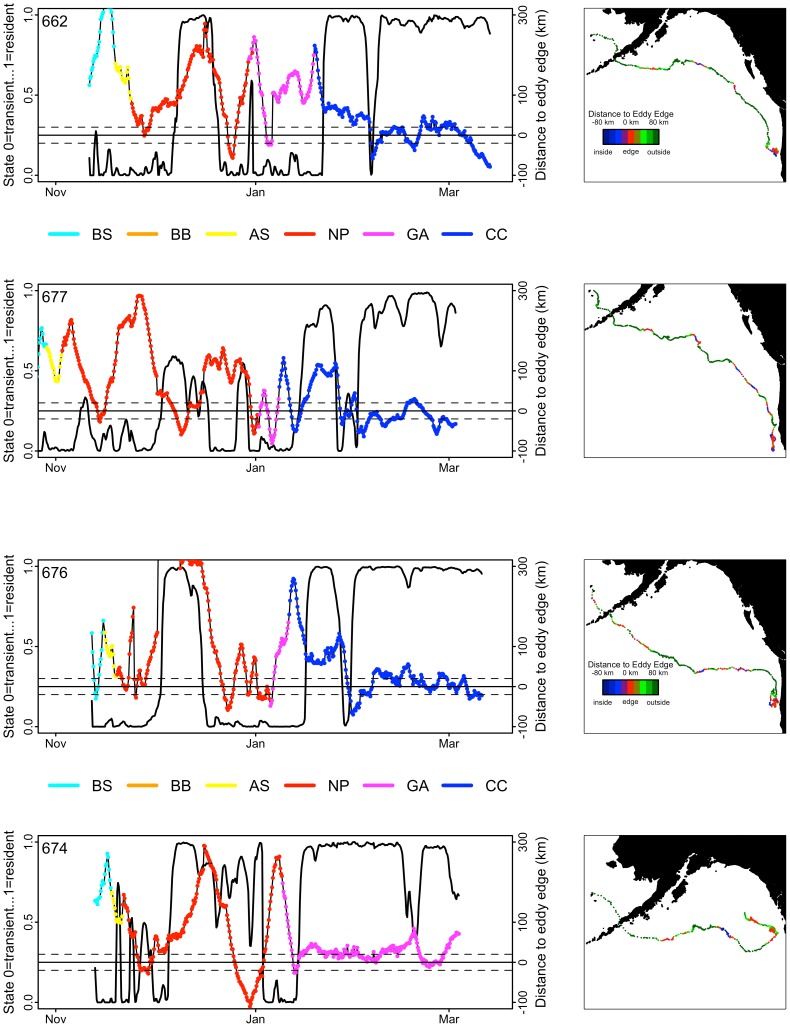
Relationship between adult female northern fur seal behavioral state, eddy distribution and ecosystem. Left panels: Behavioral states (solid black line; left y-axis) of four females compared to the distance of the closest eddy edge (colored dots; right y-axis) in each Large Marine Ecosystem traversed: NP = North Pacific Ocean, AS = Alaska Stream, BB = Bering Sea Basin, BS = Bering Sea Shelf, CC = California Current, GA = Gulf of Alaska (see Fig. 1). Right panels: female geographic distributions–color dots scale distance to the closest eddy edge. While females traversed several eddies during their winter migration, they appeared to dramatically shift their behavioral state to resident when aligned along eddy edges and once they arrived in the GA and CC ecosystems.

The spatial gradient from many to few stormy days across the range of the animals tracked in this study showed that adult males foraged in areas experiencing high wind speeds, consistently, throughout November-March (Fig. 7). Unlike males, adult females continued to migrate south and east to ecosystems experiencing a lower proportion of stormy days (Fig. 7), all the while transitioning from transient to resident behavioral states (Fig. 8 left panels). The transition coincided with their proximity to mesoscale eddies located in known energetic regions of the GA and CC (Fig. 8). Interestingly, these were not the first eddies each of these females encountered along their migratory route. For each of the females shown in Figure 8, encounters with mesoscale eddies were common, but consistently passed by when the encounters occurred in the NP and in areas experiencing high wind speeds (Figs. 7 and 8).

## Discussion

The annual migration of adult NFS from St. Paul Island begins in late autumn (October-November) and ends in late spring and early summer (May-July) when animals return to the island to reproduce and forage in the Bering Sea. Seasonal changes in the ocean and atmosphere, in particular the strong northerly winds of autumn cyclones, influence the migratory season with early storms causing earlier onset of migration than when autumn storms begin later [31], [52], [53]–10 of the 15 seals in this study departed during periods of high wind speeds generated by two autumn cyclones passing by St. Paul Island. The migration route begins with adult females departing the rookeries, traveling south through passes in the eastern Aleutian Islands, then across the NP to the continental margin of northwestern North America or the TZCF, as shown in this study and previously by [15], [16], [29]–[31]. In contrast, adult males are distributed in an expansive region of the northern NP, Aleutian Islands, GA, and Bering Sea throughout the entire winter and spring months, as also shown in this study and by [29], [31], [32].

### Adult Female Migratory Behavior

The females in this study exhibited typical migration patterns–five of six with longer lasting tags (>60 d) departed to the southeast, exited the Bering Sea through Unimak Pass, and continued south and east across the NP. The other female briefly swam northwest of St. Paul Island before reversing direction and exiting the BS. Three of the six females arrived at their destinations in the CC, a fourth nearby in the southern GA region, and the remaining two farther north in the GA. Their behavioral states across the NP were influenced by wind speed and season–females traveled more (transient state) and with the wind (not shown) during high winds and travelled less (resident state) during low to moderate winds (Fig. 3). The seasonal shift in movement behavior from transient to resident occurred upon the arrival of females in the GA and CC, regions previously identified as destination areas [16], [31]. We believe the shift in movement reflected arrival in a more equitable foraging environment that was less disrupted by strong North Pacific gales (Fig. 7). This is important in its own right, but wind also is important to the vertical distribution of biomass in mesoscale eddies, which are well known to be pelagic oases important for energy transfer to higher trophic level species [13], [54]–[56] and which are targeted by females once they reach the GA and CC (Fig. 8).

Mesoscale eddies are ubiquitous across the migration route of females from the BB to the GA and CC [51], [57] yet they appear to have little effect on female behavioral state when transiting the NP. The differential influence of eddies on female behavior might be related to female migratory momentum across the NP when they are prone to remain in transit mode to their destinations in the GA and CC, and once there enter into feeding mode, particularly around the periphery of eddies. However, the behavioral responses, or lack of responses, of females to eddies might be caused by a broader, multi-trophic level dispersion or aggregation of food web species within the females’ foraging depth range. In the case of high winds interacting with eddies, Mackas et al. [13] concluded that aggregation and retention of zooplankton were linked to behavior in the water column, and the most successful taxa migrated below the MLD to minimize exposure, thus avoiding wash out due to an eddy “spinning down” or decaying (also described by Bakun [58]), Ekman transport during strong wind events, or displacement caused by wind-driven inertial currents. The depth range of zooplankton predators, such as squid and myctophids, thus would reflect the upper depth range of their prey during diel vertical movements and would be more or less accessible to female fur seals depending on the depth. Females in this study passed through several eddies during their transit across the NP (Fig. 8), particularly in regions experiencing high winds (Fig. 7). However, upon their arrival in the GA and CC, regions that experienced lower wind speeds, females transitioned from a transitory to a more resident behavioral state in association with eddies (Fig. 8). Under the Mackas et al. [13] scenario, eddies in the stormy regions far offshore may have enhanced biological aggregations, but the vertical distribution of fur seal prey fields may be too deep during the day and too dispersed at night for successful foraging. In contrast, eddy and storm interactions were greatly diminished along the continental margins of western North America, potentially allowing for shallower and more accessible prey fields that lead to the significant shifts in female movement and dive behavior.

Females foraged predominantly at night in the upper 30 m of the water column when in the BS, NP, and GA. In contrast, the female with a dive satellite tag that travelled to the CC foraged less at night and more during the day after arriving there. Nocturnal foraging is undoubtedly related to the diel vertical distribution of prey, as suggested by others based on behaviors of known vertically migrating mesopelagic prey species such as myctophids and squids [16], [44], [59]–[62]. The contrasting behavior of the female in the CC also may be related to the availability of prey–common prey of NFS in the CC in the past consisted of Pacific whiting (*Merluccius productus*), northern anchovy (*Engraulis mordax*), Pacific herring (*Clupea pallasii*), rockfish (*Sebastes* spp.), and salmon (Salmonidae) [44], [63], epipelagic species that are typically found at comparatively shallow depths both day and night. Prey distribution and fur seal behavior may be further influenced by the MLD, which in winter along the eastern boundary of the North Pacific Ocean can be as much as ∼50 m shallower than in central and western regions (see http://www.afsc.noaa.gov/Quarterly/amj2012/AMJ12_Feature-V2.pdf) [64], making it and associated prey fields more accessible to females during the day.

Day-night diving depths support our interpretation of foraging times–that is, females tended to dive deepest during the day when prey, even in the CC, are deeper in the water column. An exception occurred in the GA, where the deepest diving was during twilight and dawn. Changing light levels at dusk and dawn signal diel vertically migrating prey to either ascend or descend in the water column. The deeper dives performed by females during these periods may reflect prey reaching water column depths within their physiological dive limits. Why this pattern was not observed in the NP or CC is unknown, but one plausible explanation is that the GA is a transitional ecosystem between the NP and CC, such that prey fields are completely inaccessible during the day in the NP, marginally accessible in the GA during twilight periods, and accessible throughout the day at shallower depths in the CC where females shift to more daytime dives.

As expected, female diving depth at night was commonly, but not always, modulated by the lunar cycle, with depths greatest during times of greatest illumination (Fig. 5, Table 5). Moonlight at night is perceived by vertically migrating prey species similarly to sunlight during the day, attenuating the vertical range of their movements and forcing predators to dive deeper to access them. The exceptions to this pattern occurred in the BS, AS, and GA where there was little to no relationship between moonlight and diving depth. With respect to the BS, this could simply be a function of their departure dates relative to the lunar cycle–most females departed closer to the new moon and exited the BS before the full moon returned. This, combined with few dives and a quick exit (Table 1), reflects the lack of foraging and thus the lack of influence of prey behavior on female behavior.

Although we obtained only a small amount of diving data from females relative to males in the NP, where we were able to determine the MLD from the male CTD records, female daytime dive depths were greater in January than in the two previous months in accordance with a deepening MLD. Data from all females tracked since 2002 [16] indicate that their daytime dive depths during fall-spring in the CC, and thus the vertical distribution of prey fields, are indeed influenced by the MLD and seasonally deepen then shoal with changing MLD (http://www.afsc.noaa.gov/Quarterly/amj2012/AMJ12_Feature-V2.pdf).

### Adult Male Migratory Behavior

Movement patterns and wintering distribution of males contrasted sharply with those of females. Males exhibited a wide variety of behaviors, from remaining in the northwestern BS near the advancing ice front throughout most of December before moving south into the NP, to swimming back and forth between the BS, BB and NP, to departing directly south and into the NP. Though all males ended up in widely separate regions, none of them ended up as far to the east as the females. Like females, their behavioral state was significantly affected by season and by high wind speeds throughout the migration (Table 3, Fig. 3). However, both the intercept and slope with respect to behavioral state differed from that of females (Table 3). Males were generally in a less transient state initially and the slopes of the seasonal effects differed from females by showing a more gradual shift to resident behavior as the season progressed (Fig. 3). The difference in the slopes between males and females could be due to males remaining in the stormy regions of the NP throughout the winter (Fig. 7) and frequently contending with strong gales.

Unlike females, which foraged mostly at night, males foraged predominantly during the day, with the exception of those in the BS where 73% of the dives occurred at night (Fig. 6, Table 4). Diving behavior was affected only marginally by the lunar cycle and primarily in the BS–had they fed more at night instead of during the day in other LMEs the lunar effect might have been stronger (Table 5). Males in oceanic LMEs apparently responded to the diel vertical movement of prey and dove deeper during the day than at night (Table 4). The day-night difference in mean dive depth was least in the BS (16 m) and much greater in the NP (65 m), BB (78 m), and AS (127 m). The small day-night difference in the BS likely reflects the shallow water depths on the shelf (<200 m), the ability of males to easily reach the seafloor, and the vertical distribution of their prey. While in the BS, males foraged at depths that corresponded approximately with the MLD, but that may be coincidental due to the close proximity of the MLD and the seafloor and not because there was a functional relationship between the MLD and distribution of prey fields.

**Figure 9:**
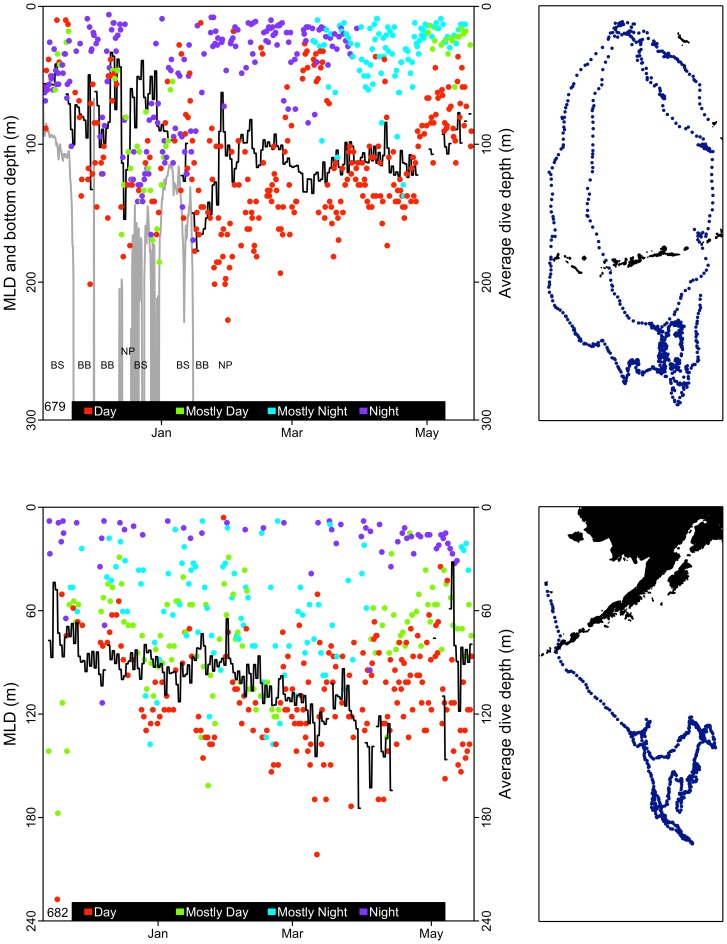
Adult male northern fur seal average dive depths in relation to the mixed-layer depth (MLD). Left panels: Average dive depth (colored dots) in relation to the MLD for males 679 and 682. The black line is the MLD calculated from the CTD measurements taken onboard the males, and the colored dots represent the proportion of light during each 6 h dive period grouped into four categories (Day, Mostly Day, Mostly Night, and Night). Male 679 (top panel) foraged in several Large Marine Ecosystems (LME) before spending the majority of his time in the North Pacific Ocean (NP) ecosystem. Each LME visited by male 679 is identified by a combination of the bottom depth (gray line) and the corresponding LME (NP = North Pacific Ocean, BB = Bering Sea Basin, BS = Bering Sea Shelf). Right panels: foraging locations of these males during the winter.

Diving depths of adult male NFS have not been reported previously. Boyd et al. [8] suggested that diving depths of adult male Antarctic fur seals, a species of similar size and life history, reflected the depth of the mixed layer. Males in all oceanic LME in this study commonly foraged to depths at and below the MLD (Fig. 9), particularly during the day. As with females in the CC (see http://www.afsc.noaa.gov/Quarterly/amj2012/AMJ12_Feature-V2.pdf), diving depths of males in the NP showed some correspondence with the seasonal deepening and shoaling of the MLD, which further suggests a functional relationship between the MLD and prey fields (Fig. 9). Male dives were correspondingly longer than female dives and males performed fewer dives per unit time (Table 4, Fig. 4).

Diets of adult males are not known for any season. Historically, adult females in winter in the NP consumed primarily squids [44], many of which are diel vertical migrants [59]–[62] and would be accessible to females foraging at night. Many dives of males went well below the MLD, and extended into the deep scattering layer (DSL) that, in winter habitat of males, lies at about 275–300 m depth [65]–[67]. Myctophids, particularly *Stenobrachius leucopsarus*, constitute a significant portion of the DSL in the NP [10] and thus would be accessible to male NFS during their deepest dives. Most squids, however, reside during the day at depths >350 m and below the presumed foraging range of males [2], [8], [26], [62], which suggests either that males are not targeting them during the day or that more is to be learned about the daytime depth distribution of squids.

### Intraspecific Competition, Differential Dietary Needs, and Predation Risk

Three factors that have been invoked to explain the differing behaviors between sexes of other species are reduced intraspecific competition, differential dietary needs due to energetic requirements, and predation risk [25]. Among pinnipeds, Breed et al. [4] believe that geographic segregation of foraging male and female gray seals (*Halichoerus grypus*) on the Scotian shelf reduces competition between them and thereby increases fitness for both sexes. For NFS, size dimorphism between the sexes is much greater compared to that of gray seals (e.g. in this study, the largest male was eight times greater in mass than the smallest female). The extreme size dimorphism and thus disparate physiological capabilities between males and females likely limits their dietary overlap and thus competition for resources. While sexual selection may be the ultimate cause of NFS size dimorphism, ecological divergence cannot be discounted. That is, adult males may gain further momentum to diverge in size with females because of the increased diving capability large size imparts, thus giving them better access to potentially abundant and energetically profitable prey resources at depth. Whichever the case, the geographic separation during the migratory period may reduce intraspecific competition for food, although there is presently no way to evaluate this possibility because of the lack of information on adult male diets in any season and location and on contemporary winter diets of adult females.

The differences in foraging strategies between sexes could be due to dietary differences owing to the physical size differential–that is, NFS males might prefer or need, because of their greater energy requirements, larger or more energy-rich prey than females – prey that might undertake attenuated or no diel vertical migration. Their much larger mouths and teeth seemingly would allow males to subdue and consume much larger prey, but again, because we know nothing about male diets, we cannot currently evaluate these possibilities.

Lastly, predation pressure has been invoked as a driver of sexual segregation in other marine mammals. Le Boeuf et al. [25] postulated that female northern elephant seals migrate to distant offshore areas of the NP to reduce the risk of predation by great white sharks (*Carcharodon carcharias*) and mammal-eating (Bigg’s) killer whales (*Orcinus orca*). The much larger males should be less susceptible to those predators in general, although proximity to both great white sharks and killer whales on their migratory routes likely increases their risk. However, if the prey resources they exploit along continental margins are more energetically profitable than those occurring in the open ocean, males may balance the risk of predation with the benefit of enhanced foraging. Another example is that of sperm whales [68], [69]: females and calves typically remain in the open NP during the summer feeding season, ostensibly to avoid killer whales, but thereby sacrificing access to the abundant prey resources enjoyed by males that migrate to the Aleutian Archipelago, BB, and GA to feed each summer and where they apparently can tolerate the risk posed by abundant killer whales. In the case of NFS the distributional patterns are reversed, making such an explanation for segregation of the sexes unlikely, since in winter the females should be more exposed to sharks and killer whales than are males. Their small size and high density along the continental margin might exacerbate the risk of predation, but because of their (former) abundance the risk to any individual might be minimized. On the other hand, one of their principal potential predators, great white sharks, is likely not abundant in winter in the colder northern part of its overall range [70], and the other, mammal eating killer whales, may not be a threat either as they typically are found in inshore areas on the continental shelf [71].

## Conclusions

Contrasting winter migration behaviors of adult male and female NFS have been documented for over a century and likely known for millennia [16], [28]–[32], [72]. For example, Aleut knowledge of differing ecosystem preferences of adult male and female NFS and effects of wind on movement patterns were reported by Captain Hooper [31] and his interpreter Peter Shainsnakoff after they interviewed 80 hunters in the Aleutian Islands during the fall of 1892. The results from our study confirm the traditional ecological knowledge told to Captain Hooper and extend this and more recent scientific knowledge, empirically, by showing the interplay between physiology, behavior, and environment.

We conclude that adult male NFS winter in the northern NP and Bering Sea because they can. They can because of the biological imperative and their life history strategy that evolutionarily led to the large size and strength necessary to hold territories against rival males while fasting during the breeding season, thus ensuring reproductive fitness. The strong positive correlations between mass and dive depth and duration across the size range of females and males reflects a gradient in the physiological dive capacity of these seals with increasing size. The vertical segregation of foraging habitat between females and males would thus be best explained by the physiological capacity of males to access prey to depths of at least 250–350 m during daytime. By remaining in the open ocean, they reduce whatever predation risk and competition that might exist along the continental margin. Moreover, their larger mass and smaller surface to volume ratio confers a thermal advantage over females, and thus males should be better able to tolerate colder seawater temperatures of northerly latitudes [53], [73]–[75]. Finally, their strength may confer a further advantage in contending with storms and turbulent seas typical of winter at higher latitudes.

Females pursue a different strategy because they must. In the context of reproductive life history traits, females are not under the same evolutionary selective pressure for greater size because they do not compete for space or mates in rookeries. But their small size places them at a disadvantage relative to males in that they are physiologically less able to reach prey occurring at greater depths at high latitudes. Instead, they seek a more equable environment, principally along the continental margin of western North America, where storms are less frequent and intense and where their dive capabilities allow them access to a diversity of prey that are predictably abundant and apparently concentrated by physical oceanographic features, particularly eddies.
